# Differential gene expression and metabolomic analyses of *Brachypodium distachyon* infected by deoxynivalenol producing and non-producing strains of *Fusarium graminearum*

**DOI:** 10.1186/1471-2164-15-629

**Published:** 2014-07-25

**Authors:** Jean-Claude Pasquet, Séjir Chaouch, Catherine Macadré, Sandrine Balzergue, Stéphanie Huguet, Marie-Laure Martin-Magniette, Floriant Bellvert, Xavier Deguercy, Vincent Thareau, Dimitri Heintz, Patrick Saindrenan, Marie Dufresne

**Affiliations:** Institut de Biologie des Plantes, Saclay Plant Sciences, Université Paris-Sud, Unité Mixte de Recherche 8618, Orsay, 91405 France; INRA/CNRS/UEVE – URGV, Saclay Plant Sciences, 2, rue Gaston Crémieux, CP5708, Evry cedex, 91057 France; INRA-AgroParisTech, MIA 518, 16 rue Claude Bernard, Paris Cedex, 75005 France; Laboratoire d’Ingénierie des Systèmes Biologiques et des Procédés – INSA, UMR INSA/CNRS 5504 - UMR INSA/INRA 792, 135 Avenue de Rangueil, Toulouse cedex 4, 31077 France; Institut de Biologie Moléculaire des Plantes, Unité Propre de Recherche du CNRS, UPR 2357, Plateforme Métabolomique, 28 rue Goethe, Strasbourg, 67083 France

**Keywords:** Fusarium Head Blight, *Brachypodium distachyon*, Transcriptome, Metabolic profiling, Serotonin

## Abstract

**Background:**

Fusarium Head Blight (FHB) caused primarily by *Fusarium graminearum* (*Fg*) is one of the major diseases of small-grain cereals including bread wheat. This disease both reduces yields and causes quality losses due to the production of deoxynivalenol (DON), the major type B trichothecene mycotoxin. DON has been described as a virulence factor enabling efficient colonization of spikes by the fungus in wheat, but its precise role during the infection process is still elusive. *Brachypodium distachyon* (*Bd*) is a model cereal species which has been shown to be susceptible to FHB. Here, a functional genomics approach was performed in order to characterize the responses of *Bd* to *Fg* infection using a global transcriptional and metabolomic profiling of *B. distachyon* plants infected by two strains of *F. graminearum*: a wild-type strain producing DON (*Fg*don^+^) and a mutant strain impaired in the production of the mycotoxin (*Fg*don^-^).

**Results:**

Histological analysis of the interaction of the Bd21 ecotype with both *Fg* strains showed extensive fungal tissue colonization with the *Fg*don^+^ strain while the florets infected with the *Fg*don^-^ strain exhibited a reduced hyphal extension and cell death on palea and lemma tissues. Fungal biomass was reduced in spikes inoculated with the *Fg*don^-^ strain as compared with the wild-type strain. The transcriptional analysis showed that jasmonate and ethylene-signalling pathways are induced upon infection, together with genes encoding putative detoxification and transport proteins, antioxidant functions as well as secondary metabolite pathways. In particular, our metabolite profiling analysis showed that tryptophan-derived metabolites, tryptamine, serotonin, coumaroyl-serotonin and feruloyl-serotonin, are more induced upon infection by the *Fg*don^+^ strain than by the *Fg*don^-^ strain. Serotonin was shown to exhibit a slight direct antimicrobial effect against *Fg*.

**Conclusion:**

Our results show that *Bd* exhibits defense hallmarks similar to those already identified in cereal crops. While the fungus uses DON as a virulence factor, the host plant preferentially induces detoxification and the phenylpropanoid and phenolamide pathways as resistance mechanisms. Together with its amenability in laboratory conditions, this makes *Bd* a very good model to study cereal resistance mechanisms towards the major disease FHB.

**Electronic supplementary material:**

The online version of this article (doi:10.1186/1471-2164-15-629) contains supplementary material, which is available to authorized users.

## Background

Among diseases affecting small-grain cereals, Fusarium head blight (FHB), mainly caused by *Fusarium graminearum* (*Fg*), is a major concern (for a review, see [1]): it reduces yields but also causes quality losses due to the production of mycotoxins, including deoxynivalenol (DON), which are harmful to humans and animals [2, 3]. *Brachypodium distachyon* (*Bd*) is an emerging model species for small-grain cereals [4]. This species is more closely-related to barley and wheat than rice and is very amenable to functional studies in laboratory conditions: a short life cycle, a small size, a routine genetic transformation and numerous genetic and genomic resources [5]. Recently, *Bd* has been shown to behave as a host plant for *Fg* [6], exhibiting characteristic symptoms, in particular at the spikes’ level, and has been proposed as a valuable model for undertaking functional genomics studies of FHB. There is no specific resistance to *Fg,* but a number of quantitative trait loci have been identified [7], some of which have been associated with the ability to detoxify DON [8, 9]. Detoxification processes in plants involve chemical modifications of the xenobiotic by enzymes such as UDP-glycosyltransferases (UGTs), glutathione-*S*-transferases (GST) or cytochrome P450 mono-oxygenases (CYP). Chemically modified metabolites are then transported either to the vacuoles or to the plant cell wall, or exported in the apoplasm [10]. A number of more or less exhaustive transcriptomic analyses conducted in bread wheat or barley have shown that genes encoding such functions were highly induced by DON-producing strains of *Fg* [11–13] and/or in resistant lines [8, 9, 14, 15]. Similarly, metabolomic studies have allowed the identification of resistance-associated metabolites, including DON-3-*O*-glucose (DON-Glc) [16, 17]. Despite these numerous studies, no direct evidence of the relationship between mycotoxin detoxification and resistance towards *Fg* has been reported to date. The main reason is that functional analyses in cereal crops are hampered by the complexity of these species.

In the present study, we aimed at better characterizing the *Bd-Fg* interaction in order to (i) further establish *Bd* as a good model system to perform functional genomics studies of the interaction; (ii) decipher the potential role of DON in the infection process and (iii) identify some potential resistance mechanisms to FHB. For this purpose, we first performed an in depth analysis of the behavior of *Bd* ecotype Bd21 plants following infection with either the DON-producing (*Fg*don^+^) or DON non-producing (*Fg*don^-^) strains of *Fg*, using a number of quantitative and qualitative traits*.* We then acquired transcriptomic and metabolomic data on the same biological material. The results are described and the main pathways/functions involved in the plant-pathogen interaction, and in response to DON are presented. The analogy to results previously described in cereal crops is discussed.

## Methods

### Plant growth conditions

*B. distachyon* ecotype Bd21 (hereafter referred to as Bd21) was cultivated in a growth chamber under a 20 h light period at 23°C ± 2°C under fluorescent light (265 μE.m^-2^.s^-1^ at the soil level and approximately 315 μE.m^-2^.s^-1^ at the spikes’ level). Prior to sowing, seeds were surface sterilized by incubation in a 0.6% sodium hypochlorite solution for 10 min with gentle shaking followed by three rinses in sterile distilled water. Sterilized seeds were subsequently incubated for five days at 4°C in the dark. Plants were grown routinely on a 3:1 mixture of compost (Tref terreau P1, Jiffy France SARL, Trevoux, France) and standard perlite (Sinclair, Gainsborough, UK), soaked with an aqueous solution containing a carbamate fungicide (Previcur at 2 ml/L, Bayer Crop Sciences, Lyon, France) and a larvicide (Hortigard at 1 g/L, Syngenta France, Guyancourt, France). Plants were usually watered in two- to three-day intervals using a standard nutritional solution and were never allowed to stand in water.

### *F. graminearum*strain maintenance and conidia production

*F. graminearum* strains PH-1 (*Fg*don^+^) and *Δtri5* (MU102 mutant strain or *Fg*don^-^, [18]) were maintained on PDA plates. To obtain conidia, 2–4 mm^2^ plugs from 15-day old PDA plates were inoculated in liquid mung bean medium ( [19], 10 plugs for 20 ml) and incubated with shaking at 150 rpm at room temperature for five to six days. The resulting conidial suspension was then diluted ten times in fresh liquid mung bean medium and further incubated for five to six additional days under the same conditions. For pathogenicity assays, conidia were further filtrated onto sterile Miracloth (Calbiochem, Toulouse, France) and resuspended in 0.01% Tween 20 at a final concentration of 10^5^ conidia/ml.

### Serotonin effect on *Fusarium graminearum in vitro*

The effect of serotonin on radial growth was estimated by placing a mycelial plug (3 mm diameter) of the *Fg*don^+^ strain in the center of a Petri dish (5.5 cm) containing PDA broth added with 0, 1 or 5 mM of serotonin (Sigma-Aldrich, Lyon, France). Radial growth was estimated after 24 and 48 h incubation at 26°C. To measure the impact on fungal sporulation, 1 ml of mung bean liquid medium containing a final concentration of 0, 1 or 5 mM serotonin was inoculated with 10^4^ conidia of the *Fg* DON^+^ strain and conidia were counted daily using a Thoma cell during one week. Conidia germination was performed on water agar (2%) containing serotonin (0, 1 or 5 mM). For each counting, the proportion of germinated conidia was estimated over a minimal total number of 100 conidia. Counting was performed 3 times for each serotonin concentration and for each incubation time after depositing conidia on the agar (3, 6 or 9 h).

### Pathogenicity assays

Inoculation was performed by depositing 300 conidia (3 μl of a 10^5^ conidia/ml suspension) into a central floral cavity of the second spikelet starting from the top of the spike of Bd21 plants at mid-anthesis (approximately 30 to 35 days after sowing). A single spike was inoculated per plant to further guarantee the independency of the samples. Inoculated plants were covered with clear plastic bags sprayed with distilled water beforehand. The inoculated heads were first kept in the dark for 24 h then incubated in a 16 h light/8 h darkness photoperiod at 20°C with light intensities similar to the ones used for plant development (see section Plant growth conditions). Application of 0.01% Tween 20 was performed as a control condition.

### Microscopy analysis

Infected spikelets were cleared in ethanol/acetic acid (3:1) 48 and 72 h after inoculation with *F. graminearum* and were stained with Trypan blue in lactophenol (Fluka, Lyon, France) following a procedure adapted from Cao et al. [20]. Spikelets were boiled for 5 min in lactophenol/Trypan blue (0.1%) and destained for 24 h in chloral hydrate solution (8 g of chloral hydrate, 1 ml 100% glycerol, and 2 ml sterile water) to remove staining. Images were captured on an Axioskop microscope (Zeiss) with a Spot RT slider camera (Diagnostic Instrument).

### Quantification of fungal genomic DNA in infected spikes

DNA was extracted from 100 mg of crushed infected spikelets. 600 μl of buffer extraction (Buffer 10x (NaCl 3.5 M, Tris HCl, pH 7.6 0.1 M, EDTA 10 mM), urea 4.2 g, EDTA 0.5 M 1 ml, phenol pH 7.5 0.65 ml and volume brought to 10 ml with water) were added then heated 5 min at 50°C and agitated 10 min at room temperature. 700 μl of phenol-chloroform-isoamyl alcohol (25-24-1) were added, the aqueous phase was extracted, 700 μl of chloroform were added and the aqueous phase was extracted again. Nucleic acids were precipitated with one volume of isopropanol and washed with 1 ml of ethanol 70%. The pellet was resuspended in water with RNase A (40 μg/ml) (Sigma Aldrich, Lyon, France). Quantification of fungal DNA was realized by qPCR on 10 ng of total DNA using primers specific for the 18S ribosomal subunit-encoding genomic region [21] (Additional file 1).

### Quantification of fungal sporulation on infected plant tissue

7, 10, 14 and 21 days after inoculation, 5 spikes (with three spikelets among which the central one was inoculated by the point inoculation method described above) were pooled in 10 ml of sterile water and shaken for 30 min at 180 rpm. They were filtrated onto sterile Miracloth (Calbiochem, Toulouse, France) and conidia were counted in a Thoma cell.

### Detection of fungal colonization of the rachis

Point inoculated spikes were collected at 7 days after inoculation. Spikelets were removed and the surface of the rachis was sterilized with ethanol 100%. Five fine slices (named a to e) from the rachis were cut and deposited onto a Petri dish containing water/agar (2%). 48 or 72 h after incubation at 26°C, the presence or absence of mycelial growth on the Petri dish out of the different rachis sections was observed.

### Material preparation for microarray experiments and transcriptome analyses

Three independent biological replicates differing by their sowing dates were produced. For each biological repetition, four inoculated spikelets per time point were collected at 0, 24, 48, 72 and 168 h after fungal inoculation (hai, strains *Fg*don^+^ or *Fg*don^-^). For the 96 hai used for the microarray analysis, 6 spikelets, coming from 6 independent plants, were collected from each biological replicate and pooled for RNA extraction. For each sample, spikelets were ground in liquid nitrogen and total RNA was extracted from 0.1 g of the resulting powder using TRIzol® reagent (Invitrogen, Life Technologies SAS, Saint-Aubin, France) followed by an RNase-free DNase I step (Ambion®, Applied Biosystems, Courtaboeuf, France) according to manufacturers’ instructions. Total RNA was subsequently cleaned using the NucleoSpin RNA Clean-up XS kit (Macherey-Nagel, Hoerdt, France) according to the supplier’s conditions.

### Microarray experiments

#### AFFYMETRIX array hybridization

RNA samples originating from the 96 hai time point were checked for their integrity on The Agilent 2100 bioanalyzer according to the Agilent Technologies (Waldbroon, Germany).

For each sample, 50 ng of total RNA was used as a manufacturer recommendation to synthesize cDNA with the Ovation® Pico WTA System V2 kit (NuGEN Technologies Inc.). Single stranded cDNA was quantified with NanoDrop® (Spectrophotometer ND1000, Thermo Fisher Scientific) and 5 μg of SPIA cDNA were used to synthesize fragmented labeled single-strand cDNA with The Encore Biotin Module kit (NuGEN Technologies Inc.).

For all samples, hybridization was performed for 16 hours at 45°C to Affymetrix GeneChip® Brachypodium Genome Array. After hybridization, the arrays were washed with 2 different buffers (stringent: 6X SSPE, 0.01% Tween-20 and non-stringent: 100 mM MES, 0.1 M [Na^+^], 0.01% Tween-20) and stained with a complex solution including Streptavidin R-Phycoerythrin conjugate (Invitrogen/molecular probes, Carlsbad, CA) and anti Streptavidin biotinylated antibody (Vectors laboratories, Burlingame, CA). The washing and staining steps were performed in a GeneChip® Fluidics Station 450 (Affymetrix). The Affymetrix GeneChip® Brachypodium Genome Arrays were finally scanned with the GeneChip® Scanner 3000 7G piloted by the Command Console Launcher Tool.

#### Statistical analysis of microarray data

The statistical analysis was based on probesets corresponding to the 53,263 genes. The data were normalized with RMA method [22] by using RMAExpress. To determine differentially expressed genes, we performed a usual two group t-test that assumes equal variance between groups. The variance of the gene expression per group is a homoscedastic variance, where genes displaying extremes of variance (too small or too large) were excluded. The raw p-values were adjusted by the Bonferroni method, which controls the Family Wise Error Rate (FWER) [23]. A gene is declared differentially expressed if the Bonferroni p-value is less than 0.05.

#### Data deposition

All raw and normalized data are available in the CATdb database [24] (Project AFFY_BRACHY_2011_11) and in the Gene Expression Omnibus (GEO) repository at the National Center for Biotechnology Information (NCBI) [25], accession number GSE50665.

### Gene annotation

Assignment of putative function to down- or up-regulated genes was performed using the combined use of the Planet site ( http://aranet.mpimp-golm.mpg.de) and NCBI Blast [26].

### Real-time PCR

cDNA synthesis was performed on 1 μg of total RNA using the ImProm-II™ reverse transcription system (Promega France, Melun-les-Charbonnières, France) according to the manufacturer’s instructions. The resulting product was diluted 10 times in nuclease-free water. Primers were designed to amplify 27 gene transcripts, including one reference gene *Bradi4g00660* (*UBC18*) as previously determined by [27] (Additional file 1). qPCR reactions were performed on 2 μl of the diluted cDNA product using 8 pmoles of each specific primer and 10 μl of SYBRGreen Master Mix in a final volume of 20 μl. Reactions were performed in a Light Cycler LC480 Real-time PCR system (Roche Diagnostics, Meylan, France). All qRT-PCR reactions were carried out on biological triplicates, each in technical duplicate. The final threshold cycle (Ct) values were the mean of the two technical duplicates. Standard deviations were calculated using the biological triplicates. The comparative ΔΔCt method was used to evaluate the relative quantities of each amplified product in the samples. The Ct was automatically determined for each reaction by the Light Cycler LC480 Real-time PCR system set with default parameters. The specificity of the qRT-PCR reactions was determined by melt curve analysis of the amplified products using the standard method installed in the system. Negative controls were included for each primer pair and the absence of primer dimers was verified. For each gene, the corresponding real-time PCR average efficiency (*E*) in the exponential phase was calculated using the equation *E* = 10[-1/slope], applied to a dilution series of cDNA pool of all samples. The PCR efficiencies of the different primer pairs used in this study are indicated in Additional file 1.

### DON quantification by chromatography coupled to mass spectrometry

400 mg of fresh ground material was extracted with 1.6 ml of methanol/water/acetic acid (80:19.5:0.5) overnight at 200 rpm, 4°C. The supernatant was recovered and the pellet was extracted once again, each time consisting in two hours at 4°C (200 rpm) with 1 ml of methanol/water/acetic acid or 0.5 ml of methanol/water (80:20), respectively. After each extraction the supernatant was pooled with the previous ones. The whole supernatant was dried with a speed vacuum concentrator and kept until analysis. In this study, DON^13^C_15_ (Romers Lab, Tulln, Austria) was used as internal standard. Plant extracts were resuspended just before analysis in 200 μl of 80% methanol and 0.1% formic acid. The samples were then analyzed on an Ultra performance liquid chromatography coupled to tandem mass spectrometry (UPLC-MS/MS) at MS and MS/MS mode. The analysis were performed on a Waters Quattro Premier XE (Waters, Mildorf, MA USA) equipped with an Electrospray Ionisation (ESI) source and coupled to an Acquity UPLC system (Waters) with diode array detector (DAD). UV spectra were recorded from 190 to 500 nm. Chromatographic separation was achieved using an Acquity UPLC BEH C_8_ column (100 × 2.1 mm, 1.7 μm; Waters), coupled to an Acquity UPLC BEH C_8_ pre-column (2.1 × 5 mm, 1.7 μm; Waters). The two mobile phases were water (UPLC MS grade, Sigma) with 0.1% formic acid (Buffer A), and methanol containing 0.1% formic acid, (Buffer B). The gradient separation, which was performed at a flow rate of 300 μl/min, was as follows: 2 min 95% A, 10 min linear gradient from 95% A to 100% B. After washing the column for 2 min with 100% B, the buffer was set back to 95% A in 1 min and the column was re-equilibrated for 2 min with 95% A (17 min total running time). To determine DON and DON^13^C_15_ best ionization parameters, positive and negative electrospray scan modes were tested. The selected Ion recording (SIR) MS mode was then used to determine parent mass transition conditions. Daughter scan MS/MS mode was then used to determine the fragmentation pattern of the different DON molecules. The quantification of the different DON molecules were made with standard molecules injected at different concentration using the MS/MS MRM (multiple reaction monitoring) mode in order to establish standard curves for each molecules. The MRM transitions resulted from SIR and daughter scan analysis and were as follow: (DON) [M + H^+^] 297.30 > 249.19, dwell (secs) 0.100, cone Voltage 20.0, collision energy 11.0, delay (secs) 0.050; (DON^13^C_15_) [M + H^+^] 312.30 > 263.15, dwell (secs) 0.100, cone Voltage 22.0, collision energy 16.0, delay (secs) 0.050. Acquisition and analysis were performed with the MassLynx software.

### Secondary metabolite analysis

Soluble metabolites were extracted as described by Simon et al. [28]. The dried extracts were resuspended in 500 μl of a mixture of methanol–water (1:1) and 1 μl was analyzed using a UHPLC/DAD instrument (Agilent technologies 1290) with a reverse-phase C18 column (Poroschell C18 2.7 μm, 150 mm × 3 mm) maintained at 40°C. The column was eluted at the flow rate 0.7 ml/min with solvent A (water added 0.4% acetic acid) and solvent B (acetonitrile) using a multi-step linear gradient between point: 100% A at 0 min, 100% A at 1.5 min, 66% A/34% B at 20 min, 100% B at 26 min, 100% B at 27 min, 100% A at 27.1 min and 100% A at 30 min. UV spectra were recorded between 200 nm to 600 nm. The UHPLC system was coupled to a qTOF mass spectrometer (Agilent technologies 6530) equipped with an ESI source operating in positive ion mode using the optimized parameters as follows: nitrogen as nebulizing gas with a flow rate of 12 L/min and a temperature of 310°C at 40 psi, scan spectra from m/z 100 to 2000, capillary voltage 3 kV, fragmentor 120 V. The detection was made considering a mass range of 80–2000 *m*/*z*. Compounds were identified by analysis of their UV, MS and MS/MS spectra and quantified with UV chromatogram at 280 nm using MassHunter Qualitative Analysis (Agilent Technologies). For absolute quantification of serotonin, tryptophan and tryptamine, samples were analyzed by HPLC on a Symmetry 5 μm C18 column (250 × 4.6 mm, Waters Corporation). We used two methods. The first one was used for serotonin and tryptophan. Samples were subjected to HPLC with 0.1% aqueous TFA as solvent A and acetonitrile with 0.1% TFA as solvent B, at a flow rate of 1.3 ml/min with the same gradient as described by Hagemeier et al. [29]. Peak detection was performed with a DAD (200–600 nm) as part of the Waters system (Waters Corporation). HPLC chromatograms were analyzed at 280 nm using Empower Pro Software (Waters Corporation). The second method was used for tryptamine. After extraction, samples were diluted in 90% TFA 0.05% (solvent A) and 10% acetonitrile (solvent B) and analyzed by HPLC coupled to a Multi λ Fluorescence Detector (Waters2475, Waters Corporation) with an excitation wavelength set at 280 nm and fluorescence emission wavelength set at 320 nm. The column was eluted at the flow rate 1 ml/min, with a multi-step gradient: 100% A at 0 min, 88% A at 6 min, 82% A at 10 min, 42% A at 30 min, 20% A at 30.5 min, 20% A at 36 min, 100% A at 36.5 min and 100% A at 42 min.

## Results

### Histological characterization of the interaction between *Bd*and *Fg*don^+^and *Fg*don^-^strains

**Figure 1:**
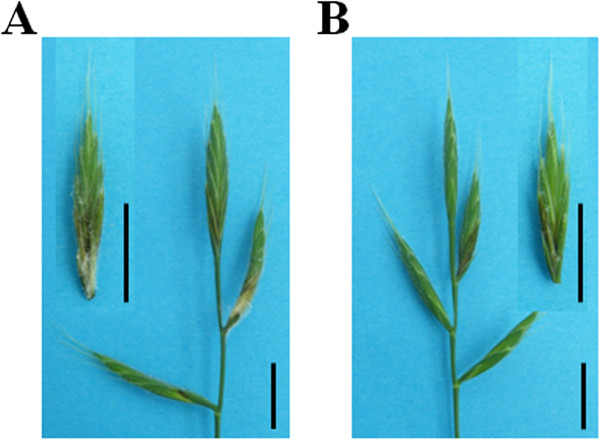
**Fusarium head blight symptoms on** ***Brachypodium distachyon*** **spikes. A**: *Fg* don ^+^ strain-induced symptoms, 96 h after point inoculation. **B**: *Fg* don ^-^ strain-induced symptoms, 96 h after point inoculation. Bars: 1 cm.

**Figure 2:**
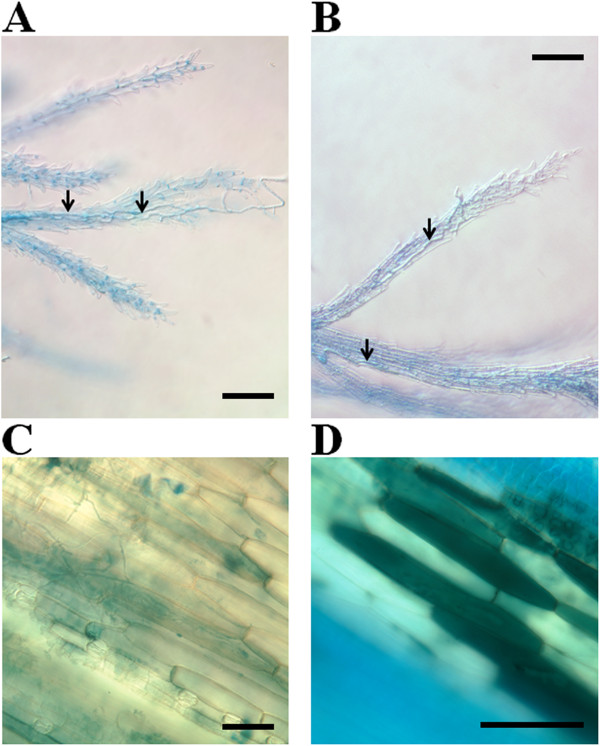
**Histology of early infection of** ***B. distachyon*** **floral cavities by** ***F. graminearum Fg*** **don** ^**+**^ **and** ***Fg*** **don** ^**-**^ **strains. A**, **B**: Penetration of feathery stigmata 24 h after point inoculation by *Fg* don ^+^ and *Fg* don ^-^ strains, respectively. **C**, **D**: Symptoms and fungal colonization on palea 72 h after point inoculation by *Fg* don ^+^ and *Fg* don ^-^ strains, respectively. Bars: 100 μm. Arrows indicate fungal hyphae.

To determine the involvement of the mycotoxin in the infection process of *Fg* on *Bd* spikes, point inoculations were performed. We used either the *Fg* wild-type strain PH-1 (*Fg*don^+^) or a mutant strain with the same genetic background impaired in the *Tri5* gene encoding the enzyme catalyzing the first committed step of the DON biosynthetic pathway, and thus unable to produce the mycotoxin (*Fg*don^-^) [18]. Following a 96 h incubation, spikes inoculated with the *Fg*don^+^ strain exhibited clear bleaching symptoms on part of the inoculated spikelet (Figure 1A) while *Fg*don^-^ inoculated plants only showed initial browning of the inoculated floral cavity (Figure 1B). Microscopy analysis showed that both strains were able to penetrate the flower through stigmata to a similar extent (Figure 2A,B). In contrast, the observation of palea tissues of the inoculated florets 72 hours after inoculation (hai) showed clear differences: while extensive fungal tissue colonization could be observed for the *Fg*don^+^ strain (Figure 2C), palea infected with the *Fg*don^-^ strain exhibited far less hyphae but numerous dying or dead cells could be observed (Figure 2D). The production of DON, the main mycotoxin synthetized by the *Fg*don^+^ strain [30], was quantified during an infection time-course. The free form of DON was detected at very low levels as early as 48 hai (1.11 ± 0.05 ppm) and then increased reaching 22 ppm (21.99 ± 1.16) at 96 hai and 51.5 ppm on average at 168 hai.

**Figure 3:**
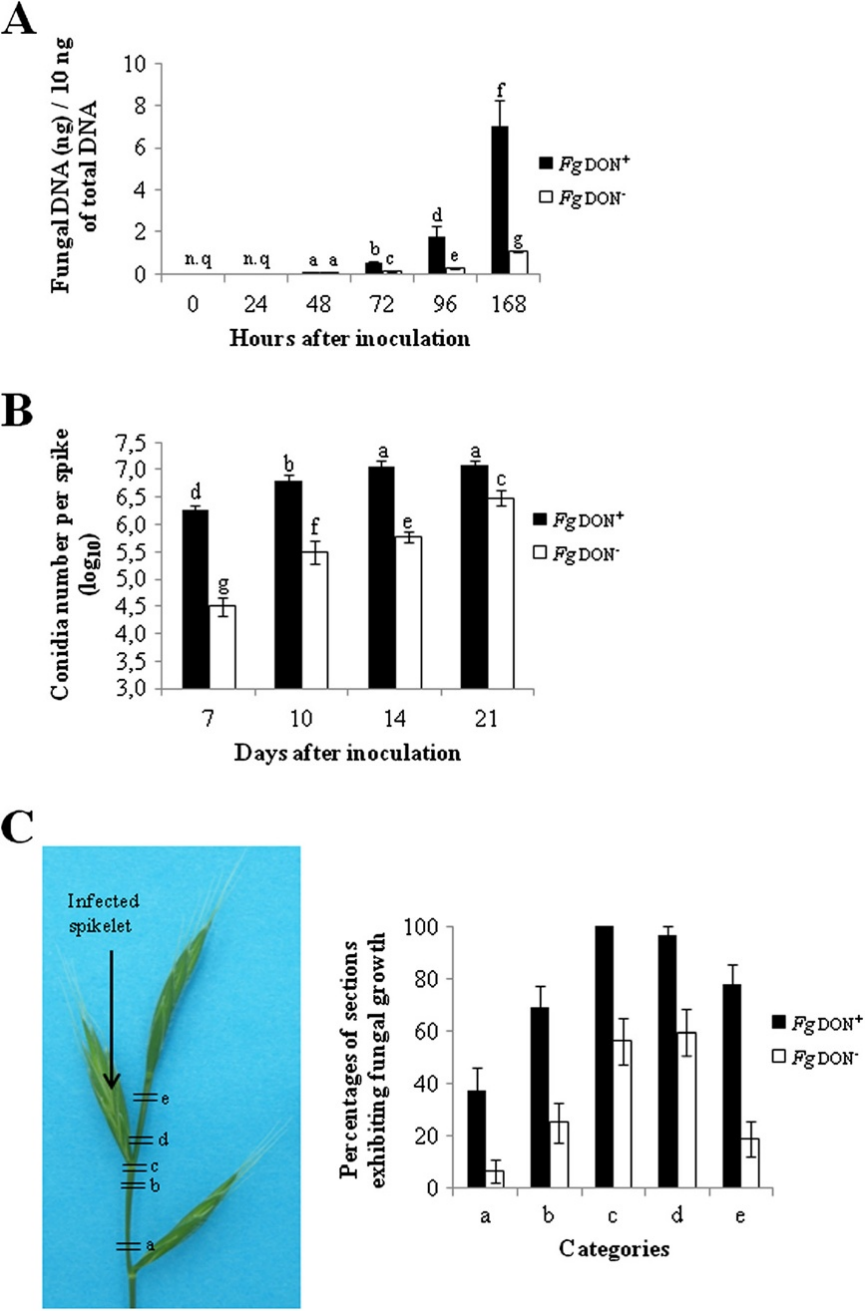
**Estimation of** ***B. distachyon*** **spike (let)s colonization of** ***F. graminearum Fg*** **don** ^**+**^ **and** ***Fg*** **don** ^**-**^ **strains** ***.*** **A**: Quantification of fungal DNA in infected spikelets (n.q. = not quantifiable, different letters indicate significant differences between conditions; t-test, p-value ≤ 0.01). **B**: Production of macroconidia on *B. distachyon* spikes infected by the *Fg* don ^+^ or the *Fg* don ^-^ strain; different letters indicate the significance of differences between conditions (Duncan test, p-value ≤ 0.01). **C**: Evaluation of *B. distachyon* rachis colonization by the *Fg* don ^+^ or the *Fg* don ^-^ strain on infected spikes collected 7 dai; left panel: localization of the different rachis sections collected, right panel: quantification of sections presenting out of which fungal growth was observed.

When incubation was extended further, the mutant strain showed a delay in colonization that tended to attenuate in the last timepoints (14 and 21 days after inoculation (dai), data not shown). In order to better compare fungal infection by the two strains than by visual symptoms assessment, the fungal biomass was quantified by measuring fungal genomic DNA by qPCR in early timepoints, until 7 dai (168 hai). As shown in Figure 3A, the amounts of fungal DNA in spikes inoculated by the *Fg*don^-^ strain were much lower than for those found in the tissues infected by the *Fg*don^+^ strain (*t* test, p-value ≤ 0.01), which well correlated with the histological observations presented above. For timepoints later than 7 dai, we quantified the ability of both strains to produce secondary inoculum, namely macroconidia, at the surface of infected spikes. These measures indicated that, even at 14 and 21 dai for which assessment of visual symptoms does not allow clear distinction between the two strains, the *Fg*don^-^ strain still exhibited a significant lower conidia production on infected plant tissues as compared with the *Fg*don^+^ strain (Duncan test, p-value ≤ 0.01; Figure 3B).

Previous studies have shown that DON enables the fungus to colonize the rachis of wheat spikes [31, 32]. To analyze the importance of DON in the colonization of the rachis, experiments were designed to detect the progress of *Fg*don^+^ and *Fg*don^-^ strains into the rachis. One-mm long transversal sections of the rachis in the vicinity of the inoculated spikelet were cut at different locations from 7 dai infected spikelet, surface sterilized and incubated on water agar for 48 h (Figure 3C, left). The presence of the fungal mycelium growing out of the sections was then recorded. Our results showed that the *Fg*don^+^ strain is able to colonize the rachis, with more efficient growth in the acropetal direction (Figure 3C, right). On the contrary, the *Fg*don^-^ strain, even if exhibiting the same acropetal preferential development, was only detected in sections in the immediate vicinity of the inoculated spikelet (Figure 3C, right).

### Transcriptome analysis of *Bd*spikes infected by *Fusarium graminearum*

To analyze the responses of *Bd* to *Fg* infection and to discriminate the responses due solely to the DON mycotoxin, a transcriptome analysis was performed using the *Fg*don^+^ and *Fg*don^-^ strains. Affymetrix GeneChip® Brachypodium Genome Array was used to examine transcript accumulation in Bd21 ecotype inoculated spikelets 96 hai by either the *Fg*don^+^ or the *Fg*don^-^ strain and compared to the mock inoculated spikelets. The 96 hai timepoint was chosen as it corresponded to the early time point which best discriminated the extent of spikelet colonization between the two strains. The analyses of the GeneChip data are presented in the Methods section. In all, 4,633 transcripts exhibiting a significant differential expression between the different conditions were retrieved (p-value ≤ 0.05 after Bonferroni correction). In the following, we limited the biological interpretation to genes exhibiting an absolute value of log_2_ fold-change equal or more than 2.0. Thus, a total of 3,506 transcripts could be classified into three categories: (1) 3,304 transcripts showed a differential accumulation between the *Fg*don^+^ strain- and mock-inoculated spikelets, (2) 596 transcripts showed a differential accumulation between the *Fg*don^-^ strain- and mock-inoculated spikelets and (3) 1,878 transcripts showed a differential accumulation between the *Fg*don^+^ strain- and *Fg*don^-^ strain-inoculated spikelets. Of the 3,304 transcripts exhibiting differential accumulation between the *Fg*don^+^ strain- and mock inoculated spikelets, 1,909 were up-regulated and 1,395 were down-regulated. Among the 596 transcripts differentially regulated between *Fg*don^-^ strain- and mock-inoculated spikelets, 555 were up-regulated and 41 were down-regulated. From the 1,878 differential transcripts detected between *Fg*don^+^ strain- and *Fg*don^-^ mutant strain-inoculated spikelets, 1,226 were up-regulated and 652 were down-regulated.

**Figure 4:**
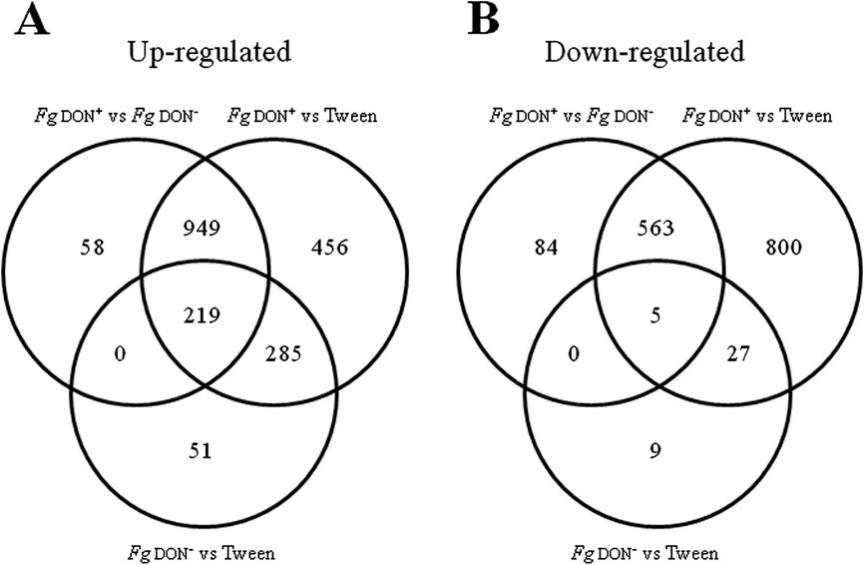
**Venn diagrams showing differentially accumulating transcripts in** ***B. distachyon*** **spikelets inoculated with** ***F. graminearum*** **wild-type (** ***Fg*** **don** ^**+**^ **) or** ***tri5*** **mutant (** ***Fg*** **don** ^**-**^ **) strains or mock-inoculated (Tween). A**: Up-regulated transcripts. **B**: Down-regulated transcripts.

To better discriminate each treatment, we examined the potential overlaps between the different conditions. The resulting Venn diagrams (Figure 4) showed that most of the differentially accumulated transcripts could be associated with the infection by the *Fg*don^+^ strain: 72.5% and 97.2% of the up- or down-regulated transcripts, respectively, were significantly more abundant in the *Fg*don^+^ strain-treated samples. 504 up-regulated and 32 down-regulated transcripts were associated with general fungal infection, independently of the fungal strain, while 1,405 genes were specifically induced by DON accumulation and 1,363 genes repressed. Finally, 60 transcripts are specific for the *Fg*don^-^ inoculated plants: 51 are up-regulated and 9 are down-regulated. To validate the microarray results, we selected twenty candidate genes and performed qRT-PCR analysis using the same RNA samples used for microarray analysis. These genes were chosen because encoding proteins potentially related to plant defense and general detoxification, but also as a representative sample of all differential regulation patterns detected following the analysis of microarray results. The results of the expression analysis strongly correlated (Table 1, R^2^ = 0.81) with the microarray data in all three treatments.

**Table 1:** **qPCR validation of selected transcripts exhibiting differential accumulation in microarray**

		Microarray		qPCR		
Gene	Comparison	Fold change (Log _2_)	***P***-val (Bonferroni)	Fold change (Log _2_)	Standard error	Gene description
*Bradi1g75310*	*Fg* don ^+^ vs Tween	6.93	0.00	14.40	0.26	Cytochrome P450 monooxygenase
	*Fg* don ^-^ vs Tween	0.77	1.00	6.28	0.40	
	*Fg* don ^+^ vs *Fg* don ^-^	6.16	0.00	8.12	0.26	
*Bradi3g22880*	*Fg* don ^+^ vs Tween	7.44	0.00	10.24	0.08	MATE efflux
	*Fg* don ^-^ vs Tween	1.36	0.18	2.26	0.11	
	*Fg* don ^+^ vs *Fg* don ^-^	6.08	0.00	7.97	0.08	
*Bradi2g41010*	*Fg* don ^+^ vs Tween	9.30	0.00	12.93	0.15	Unknown
	*Fg* don ^-^ vs Tween	4.80	0.00	3.84	0.25	
	*Fg* don ^+^ vs *Fg* don ^-^	4.51	0.00	9.08	0.15	
*Bradi2g35930*	*Fg* don ^+^ vs Tween	8.19	0.00	10.09	0.07	Glutathione S-transferase GSTF1-like
	*Fg* don ^-^ vs Tween	4.15	0.00	3.46	0.45	
	*Fg* don ^+^ vs *Fg* don ^-^	4.05	0.00	6.63	0.07	
*Bradi4g39950*	*Fg* don ^+^ vs Tween	2.38	0.00	3.12	0.19	NADP-dependent alkenal double bond reductase P1
	*Fg* don ^-^ vs Tween	5.68	0.00	6.05	0.28	
	*Fg* don ^+^ vs *Fg* don ^-^	-3.29	0.00	-2.93	0.19	
*Bradi4g05040*	*Fg* don ^+^ vs Tween	2.25	0.00	3.35	0.06	PR10
	*Fg* don ^-^ vs Tween	7.48	0.00	8.42	0.27	
	*Fg* don ^+^ vs *Fg* don ^-^	-5.22	0.00	-5.08	0.06	
*Bradi4g36870*	*Fg* don ^+^ vs Tween	3.69	0.00	4.75	0.05	Ornithine decarboxylase
	*Fg* don ^-^ vs Tween	0.90	1.00	1.59	0.50	
	*Fg* don ^+^ vs *Fg* don ^-^	2.77	0.00	3.16	0.05	
*Bradi4g36810*	*Fg* don ^+^ vs Tween	5.06	0.00	10.14	0.28	Ornithine decarboxylase
	*Fg* don ^-^ vs Tween	1.37	0.15	5.55	0.26	
	*Fg* don ^+^ vs *Fg* don ^-^	3.69	0.00	4.59	0.28	
*Bradi1g50067*	*Fg* don ^+^ vs Tween	3.63	0.00	5.23	0.16	Arginine decarboxylase
	*Fg* don ^-^ vs Tween	1.16	1.00	1.58	0.31	
	*Fg* don ^+^ vs *Fg* don ^-^	2.46	0.00	3.65	0.16	
*Bradi3g47110*	*Fg* don ^+^ vs Tween	5.86	0.00	11.99	0.34	Phenylalanine ammonia-lyase
	*Fg* don ^-^ vs Tween	0.32	1.00	5.59	0.59	
	*Fg* don ^+^ vs *Fg* don ^-^	5.53	0.00	6.41	0.34	
*Bradi3g53227*	*Fg* don ^+^ vs Tween	3.56	0.00	6.94	0.14	3-hydroxy-3-methyl-glutaryl-CoA reductase
	*Fg* don ^-^ vs Tween	0.17	1.00	1.77	0.15	
	*Fg* don ^+^ vs *Fg* don ^-^	3.40	0.00	5.17	0.14	
*Bradi2g13360*	*Fg* don ^+^ vs Tween	4.10	0.00	5.69	0.14	Mevalonate kinase
	*Fg* don ^-^ vs Tween	1.93	0.00	2.26	0.36	
	*Fg* don ^+^ vs *Fg* don ^-^	2.17	0.00	3.43	0.14	
*Bradi5g02780*	*Fg* don ^+^ vs Tween	2.41	0.00	4.87	0.16	UDP-glycosyltransferase
	*Fg* don ^-^ vs Tween	1.16	1.00	2.66	0.36	
	*Fg* don ^+^ vs *Fg* don ^-^	1.25	1.00	2.21	0.16	
*Bradi5g03300*	*Fg* don ^+^ vs Tween	7.46	0.00	12.01	0.16	UDP-glycosyltransferase
	*Fg* don ^-^ vs Tween	0.88	1.00	2.92	0.47	
	*Fg* don ^+^ vs *Fg* don ^-^	6.59	0.00	9.08	0.16	
*Bradi5g03370*	*Fg* don ^+^ vs Tween	6.61	0.00	9.51	0.19	UDP-glycosyltransferase
	*Fg* don ^-^ vs Tween	2.09	0.00	3.29	0.44	
	*Fg* don ^+^ vs *Fg* don ^-^	4.52	0.00	6.22	0.19	
*Bradi5g03380*	*Fg* don ^+^ vs Tween	6.72	0.00	12.46	0.22	UDP-glycosyltransferase
	*Fg* don ^-^ vs Tween	1.52	0.01	4.36	0.73	
	*Fg* don ^+^ vs *Fg* don ^-^	5.20	0.00	8.10	0.22	
*Bradi5g03390*	*Fg* don ^+^ vs Tween	4.34	0.00	6.23	0.08	UDP-glycosyltransferase
	*Fg* don ^-^ vs Tween	1.86	0.00	2.15	0.44	
	*Fg* don ^+^ vs *Fg* don ^-^	2.49	0.00	4.08	0.08	
*Bradi5g03400*	*Fg* don ^+^ vs Tween	1.22	1.00	2.51	0.13	UDP-glycosyltransferase
	*Fg* don ^-^ vs Tween	1.37	0.14	2.01	0.10	
	*Fg* don ^+^ vs *Fg* don ^-^	-0.16	1.00	0.50	0.13	
*Bradi2g04720*	*Fg* don ^+^ vs Tween	5.41	0.00	8.76	0.20	UDP-glycosyltransferase
	*Fg* don ^-^ vs Tween	3.22	0.00	5.47	0.24	
	*Fg* don ^+^ vs *Fg* don ^-^	2.19	0.00	3.29	0.20	
*Bradi2g04760*	*Fg* don ^+^ vs Tween	4.71	0.00	7.25	0.19	UDP-glycosyltransferase
	*Fg* don ^-^ vs Tween	1.04	1.00	2.57	0.26	
	*Fg* don ^+^ vs *Fg* don ^-^	3.67	0.00	4.68	0.19	

### Defense-related responses and signalling pathways in the *B. distachyon*-*F. graminearum*interaction

For further and more in depth analyses, we chose a gene subset consisting of 650 genes exhibiting a regulation with a log_2_ ratio equal or more than 3.0, corresponding to genes either up- (582 genes) or down-regulated (68 genes) following infection with the *Fg*don^+^ strain as compared with infection with the *Fg*don^-^ strain. A classification and more detailed functional annotation of these genes are presented in Table 2 and Additional file 2, respectively. The expression of the vast majority of these genes (459/650, 70.6%) was not significantly regulated following infection by the *Fg*don^-^ indicating that this subset is of particular interest for the analysis of DON-regulated transcripts. Among the annotated transcripts exhibiting a down-regulation after challenging with the *Fg*don^+^ strain, two main functional categories were over-represented (Additional file 2): (1) functions involved in photosynthesis (18 transcripts) and general metabolism, in particular glycolysis (4 transcripts). The annotated up-regulated transcripts fell into more diverse functional categories: plant defenses and associated signalling pathways, oxidative stress, secondary metabolism pathways, plant detoxification and signal transduction (Additional file 2).

**Table 2:** **Main characteristics of transcripts exhibiting an absolute value of log**_**2**_ **fold change ≥ 3.0 between the two infection conditions**

Functional category	Number	Percentage	Main tendancy*
Defense associated proteins	37	5.7	I (35/37)
Proteins involved in oxidative burst/redox status	17	2.6	I (14/17)
Cytochrome P450s	17	2.6	I (16/17)
UDP-glycosyltranferases	16	2.5	I (16/16)
Glutathion-*S*-tranferases	9	1.4	I (8/9)
Transport	24	3.7	I (22/24)
Amino acid metabolism	11	1.7	I (11/11)
Hormones synthesis and response	35	5.4	I (34/35)
Secondary metabolism	10	1.5	I (10/10)
Ubiquitination	12	1.8	I (12/12)
Signal transduction	96	14.8	I (95/96)
Regulatory proteins	77	11.8	I (72/77)
Photosynthesis	22	3.4	R (18/22)
Translation/Ribosomes	12	1.9	I (10/12)
Lipid metabolism	7	1.1	I (6/7)
General metabolism	7	1.1	R (5/7)
Miscellaneous	102	15.5	I (87/102)
Unknown proteins	139	21.5	I (128/139)
**Total**	**650**	**100**	

*Numbers in brackets represent the number of transcripts showing induction (I) or repression (R) among the total number of transcripts in each functional category.

Contrary to other categories, half of the defense-related genes (18/37 transcripts) were significantly induced in both infection conditions, with a higher induction following infection by the *Fg*don^+^ strain. Among these genes, a number were related to general plant defenses (Additional file 2). In addition, in this category, *MLA6* (*Bradi2g11930*), an ortholog of the barley *MLA* resistance genes [33] is noteworthy as it was only induced by the *Fg*don^+^ strain. Among the defense-associated signalling pathways, jasmonic acid (JA) and ethylene (ET) biosynthesis and signalling pathways were shown to be induced upon infection by both *Fg* strain. A putative lipoxygenase (*LOX*) gene (*Bradi5g11590*) and a gene potentially encoding a 12-oxophytodienoate reductase (*Bradi1g45880*), two enzymatic activities known to be important for oxylipins biosynthesis [34], were more strongly induced by the *Fg*don^+^ strain than by the *Fg*don^-^strain. Four genes encoding putative ZIM domain containing proteins related to JAI3 (JASMONATE-INSENSITIVE 3) [35] were also differentially induced, but only *Bradi3g23180* was specifically up-regulated by the *Fg*don^+^ strain. Moreover, potential ET-responsive transcription factor-encoding genes appeared to be induced upon infection by both *Fg* strains, but some were only related to the *Fg*don^+^ infection, such as the *Bradi2g25050* gene encoding a putative AP2/EREB (APETALA2/Ethylene Response Elements Binding) protein (Additional file 2).

Genes encoding proteins with known anti-oxidative activities were also highly induced. Among these, a number were significantly more induced following infection by the *Fg*don^+^ producing strain (Table 2; Additionnal file 3), including genes encoding a putative ascorbate oxidase (*Bradi4g28950*), 2 potential glutaredoxins (*Bradi2g08400; Bradi2g46090*) and 5 oxido-reductases. Of interest is the presence of 7 members of the cupin superfamily related to the oxalate oxidase-like proteins generating hydrogen peroxide (H_2_O_2_) [36]. The *Bradi1g59440* gene induced in both infections, encodes a putative plant flavodoxin-like quinone reductase [37]. Quinone reductases are flavoproteins that protect plants from oxidative stress induced by necrotrophic fungi [37].

Pathogenesis-related proteins encoding genes revealed a more contrasted situation, some exhibiting a higher induction, while others were clearly repressed 96 hai by the *Fg*don^+^ strain. The expression profile of a number of these genes was examined during infection time courses, from 0 to 168 hai, by either the *Fg*don^+^ or the *Fg*don^-^ strain (Additional file 3). Some genes, including those encoding a chitinase (*Bradi2g26810*) and a PR9-like peroxidase (*Bradi1g39190*), exhibited a significantly higher induction following infection by the *Fg*don^+^ strain, likely reflecting the delayed colonization of the *Fg*don^-^ strain (Additional file 3A). By contrast, others showed a higher induction by the *Fg*don^-^ strain, such as those encoding PR1 (*Bradi1g12360*) - and PR10-like (*Bradi4g05040*) proteins (Additional file 3B).

### Secondary metabolism and plant detoxification

**Figure 5:**
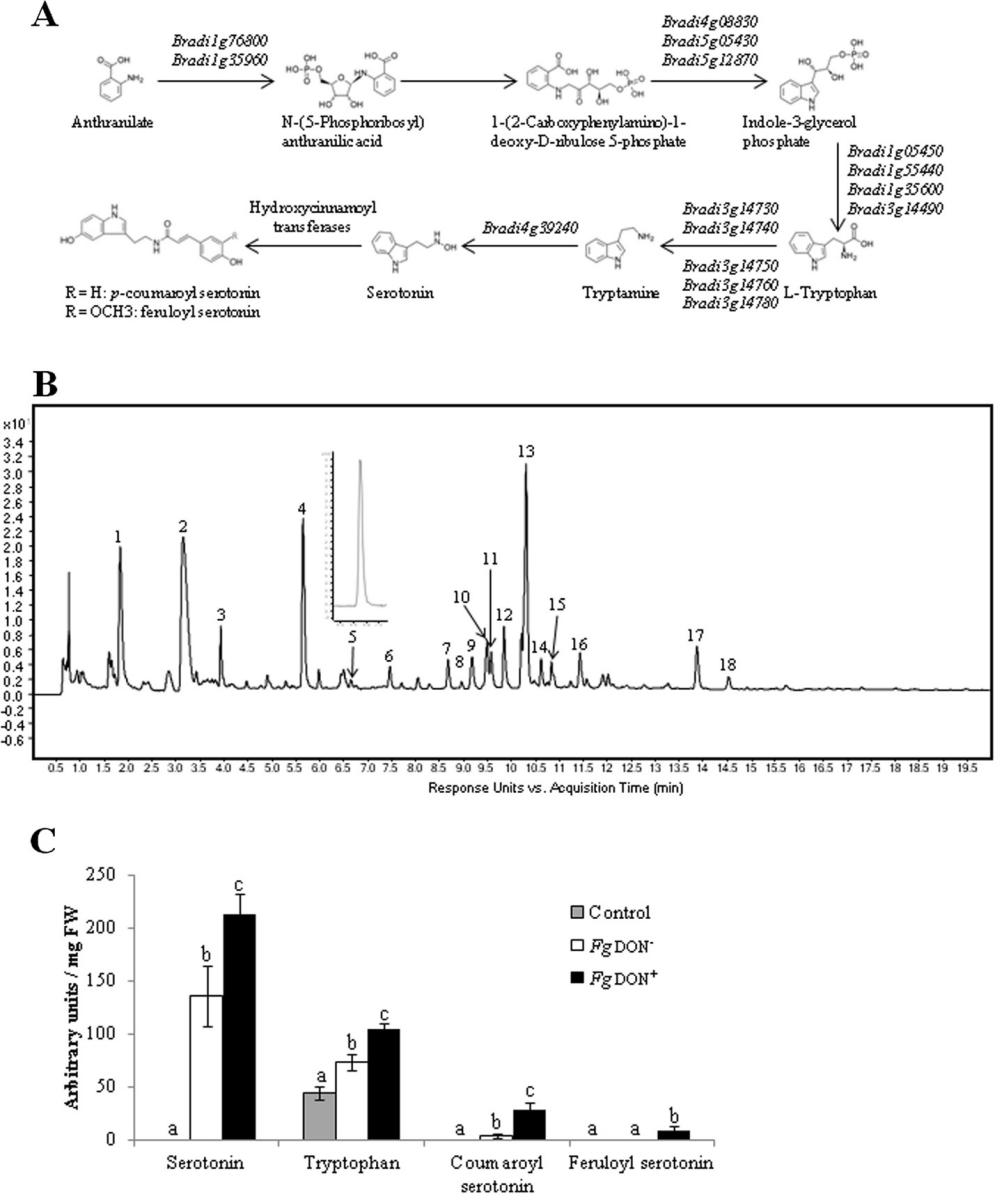
**Induction of the tryptophan pathway during the interaction between** ***B. distachyon*** **and** ***F. graminearum*** **. A**: Reconstructed tryptophan pathway in *B. distachyon*. The accession numbers of *B. distachyon* genes encoding enzymes potentially involved in the metabolic pathway and exhibiting a differential expression between the conditions used for transcriptome analyses are indicated over or next to each arrow (for more information see Additional file 4). **B**: Metabolic profiling of soluble secondary metabolites from *B. distachyon* spikelets 96 h after infection by the *F. graminearum Fg* don ^+^ strain. HPLC profiles were obtained from aqueous methanolic extracts. Chromatograms were analysed by UV detection at 280 nm. For a correspondence of peak numbers, see Additional file 5. **C**: Relative quantification of metabolites derived from the tryptophan pathway (different letters indicate significance of the difference between conditions; t-test p-value ≤ 0.02).

Most genes involved in secondary metabolism pathways or plant detoxification exhibited a differential regulation of their expression between the different treatments (Table 2), many of them being up-regulated following infection by both strains, but to a greater extent in the *Fg*don^+^ strain treatment. In our conditions, the most induced genes were involved in the phenolic biosynthetic pathways, either the phenylpropanoid or phenolamide pathways (Additional file 2). The expression of two putative *PAL* (Phenylalanine Ammonia Lyase) genes (*Bradi3g47110* and *Bradi3g49270*) was up-regulated during infection, both being specifically induced by the *Fg*don^+^ strain. A number of genes identified as up-regulated were related to amino-acid metabolism, in particular to the tryptophan (Trp) metabolism (Table 2 and Additional file 2). Functional annotation using the KEGG (Kyoto Encyclopedia of Genes and Genomes) database allowed us to identify genes potentially encoding the enzymes in the metabolic pathway from anthranilate to serotonin (Figure 5A and Additional file 4) as those described in rice infected by *Bipolaris oryzae* [38]. More precisely, 4 genes encoding proteins related to the putative Trp decarboxylase (TDC) enzymes (*Bradi3g14730*; *Bradi3g14760*; *Bradi3g14750*; *Bradi3g14740*) and a gene encoding a putative indole-3-glycerol phosphate lyase (*Bradi1g05450*) were more induced after infection with the *Fg*don^+^ strain compared to the infection with the *Fg*don^-^ strain (Additional file 4). Particularly interesting was the *Bradi4g39240* gene encoding a cytochrome P450 monooxygenase (CYP71P1) that catalyzes the conversion of tryptamine to serotonine in rice [39]. Finally, our data from the Additional file 2 pointed to 5 genes encoding putative hydroxycinnamoyl transferases classified into the phenolamide biosynthesis category. No genes belonging to the benzoxazinoid pathway could be retrieved from our transcriptomic analysis.

We then performed a metabolic profiling analysis of soluble metabolites on the same biological samples used for microarray and qRT-PCR analyses. A typical chromatogram obtained from *Fg*don^+^ strain-infected plant tissues at 96 hai is shown in Figure 5B. In this chromatogram, 18 peaks could be clearly individualized (Additional file 5). Among these, 5 peaks exhibited significant difference both with the control samples and between the two infection treatments (*t* test, p-value ≤ 0.02). These peaks could be identified as Trp (peak 4) or Trp-derived compounds: tryptamine (peak 5), serotonin (peak 2) and coumaroyl (Cou)- and feruloyl (Fer)-serotonin (peaks 17 and 18, respectively). In order to compare the two infection treatments and the mock condition, relative quantifications were conducted on all compounds except for tryptamine, for which quantities were too low to be detected to perform proper relative quantification. As shown in Figure 5C, all compounds accumulated following fungal infection, with higher increase folds for serotonin and Cou-serotonin. The higher increase was observed following infection by the *Fg*don^+^ strain, while the *Fg*don^-^-infected spikelets also exhibited an increased amount of the four compounds but to a lesser extent. Trp, tryptamine and serotonin absolute quantification was performed using UV-fluorescence detection. This analysis confirmed the previous results, showing an increased accumulation of the three compounds in infected spikelets, but with a significant difference between *Fg*don^+^ and *Fg*don^-^ infected plants (Additional file 6).

A number of genes differentially induced in the *Fg*don^+^ infected spikelets were functionally related to general detoxification [10] (Table 2, Additional file 2). These included genes encoding UDP-glycosyltransferases (UGT, 16 genes), glutathione-*S*-transferases (GST, 8 genes), cytochrome P450 monooxygenases (CYP, 16 genes) as well as transporters such as pleiotropic drug resistance, ATP-Binding Cassette (ABC) and Multidrug And Toxic Compound Extrusion (MATE) transporters (13 genes). More than 50% of these genes (31/53) showed no significant differential expression in the *Fg*don^-^ strain-infected spikelets (Bonferroni correction, p-value > 0.05) suggesting their potential role in mycotoxin detoxification. In particular, the *Bradi3g31860* gene encoding a GST was specifically induced upon infection with the *Fg*don^+^ strain. Interestingly, among the UGT-encoding genes, the most induced gene (*Bradi5g03300*) exhibiting a log_2_ fold change of more than 6.5 between the two infection conditions encodes a UGT already shown to be able to detoxify DON into DON-glucose [40]. In contrast, a gene encoding a NADP-dependent alkenal double bond reductase, an enzymatic activity previously shown to be involved in the detoxification of reactive carbonyls in plants [41], exhibited a higher induction by the *Fg*don^-^ strain (Additional file 3B).

### Antimicrobial activity of serotonin against *Fusarium graminearum*

Serotonin has been reported in several plant species and associated with different processes including defense responses [38, 42]. Recently, the production of serotonin has been shown to be induced in wheat by the toxin SnToxA from *Stagonospora nodorum* and serotonin has been shown to have antimicrobial properties [42]. To determine if this compound exhibited antimicrobial activities against *F. graminearum*, serotonin was applied *in vitro* at different developmental stages of the fungus. Two concentrations were used, 1 and 5 mM, which encompassed the amount of serotonin detected in infected spikelets (Additional file 6). As shown in Table 3, the higher serotonin concentration slightly reduced radial growth (Duncan test, p-value ≤ 1.10^-4^) as well as delayed conidia germination (*t* test, p-value ≤ 0.01). In contrast, both concentrations of serotonin seemed to increase conidia production in liquid mung bean medium.

**Table 3:** **Serotonin effect on radial growth, conidia production and germination of** ***Fusarium graminearum*** **(strain** ***Fg*** **don** ^**+**^ **)**

		Serotonin concentration		
		0 mM	1 mM	5 mM
Radial growth	24 h	2.58 ± 0. 07^c^	2.64 ± 0.11^c^	2.23 ± 0.07^d^
	48 h	4.81 ± 0.11^a^	4.84 ± 0.12^a^	4.43 ± 0.09^b^
Conidial concentration (log_10_)	48 h	5.60 ± 0.12^i^	5.94 ± 0.04^gh^	5.95 ± 0.03^gh^
	72 h	5.82 ± 0.08^hi^	6.21 ± 0.02^ef^	6.16 ± 0.08^ef^
	96 h	5.93 ± 0.07^gh^	6.26 ± 0.05^de^	6.26 ± 0.06^de^
	120 h	6.09 ± 0.08^fg^	6.32 ± 0.07^cd^	6.42 ± 0.04^ab^
	144 h	6.18 ± 0.07^ef^	6.37 ± 0.06^bc^	6.45 ± 0.03^a^
	168 h	6.22 ± 0.04^e^	6.41 ± 0.05^ab^	6.47 ± 0.03^a^
Spore germination (percentage)	3 h	72.61 ± 5.02^b^	72.87 ± 5.12^b^	25.42 ± 2.02^c^
	6 h	93.97 ± 2.35^a^	91.7 ± 2.12^a^	79.01 ± 1.41^b^
	9 h	98.55 ± 0.95^a^	97.35 ± 1.51^a^	92.81 ± 1.68^a^

Different letters indicate significance of the difference between conditions; Duncan test, p-value ≤ 0.01.

## Discussion

*Bd* has emerged recently as a novel monocot model plant species in the last 15 years for functional genomics approaches in small-grain cereals [4, 5, 43]. The shortness of its developmental cycle [44], together with the amenability of genetic transformation [45–48] and the availability of mutant collections [49–52] and of the genome sequence [53] makes this plant species an easy model to work with as a host for the interaction with important cereal pathogens such as *Magnaporthe grisea* [54], *Pyrenophora teres* [55], *Puccinia graminis* [56], the *Panicum mosaic virus* [57] and *Fusarium* species [6, 58]. This is of particular interest since this allows functional genomics studies to be performed to better understand cereals resistance mechanisms to important diseases. In the present study, we were interested in Fusarium Head Blight, one of the major small-grain cereals’ diseases [59]. The objective was to gain insight into the *Bd*-*Fg* interaction using high throughput methods.

We first characterized the interaction of *B. distachyon* ecotype Bd21 with two *F. graminearum* strain: a wild-type strain PH-1, *Fg*don^+^, producing deoxynivalenol (DON) and a near-isogenic strain, *Fg*don^-^, unable to produce the mycotoxin, following inactivation of the gene encoding the trichodiene synthase, the first enzyme of DON biosynthetic pathway [60]. DON accumulated to similar levels in inoculated spikes as those detected in wheat and barley infected by *Fg* [11, 14]. We showed that overall, the absence of DON production leads to a dramatically delayed infection, exemplified by a strong decrease of both the fungal biomass *in planta* and the production of secondary inoculum over infected heads by the mutant strain as compared with its wild-type relative. The infection delay can be explained by the impairment of the mutant strain in rachis colonization. Even if less clear-cut, our observations are highly reminiscent to the situation in wheat in which fungal strains impaired in mycotoxin production were shown to be blocked at the initial inoculated spikelet, due to the development of important cell-wall thickenings at the rachis node [31, 32]. The delay rather than complete blocking of fungal colonization from the inoculation site is most likely due to the size and architecture of *Bd* spikelets and spikes. Peraldi et al. [6] also showed that *Bd* was susceptible to *Fg* and *Fusarium culmorum* in a similar manner to that observed in wheat, but the authors did not provide conclusive evidence on the role of DON in fungal virulence. Here we genetically demonstrated that *Fg* uses its main mycotoxin, DON, as a virulence factor during *Bd* infection.

While resistance to Fusarium Head Blight in wheat and barley is quantitative [7, 61], so far, no quantitative resistance traits to *Fg* have been described in *Bd*. Thus, we performed an RNA profiling approach to examine the *Bd*-*Fg* interaction. 1,878 transcripts differentially regulated in the two infection conditions were identified. Most of them were shown to be associated with the infection with the *Fg*don^+^ strain, a condition correlated with the production of mycotoxin and with a more intense fungal colonization of the inoculated spikelets. For 20 of these genes, a comparative qRT-PCR analysis confirmed the transcriptomic data.

To better gain insight into the defense-associated and signalling pathways, and functions differentially regulated between the two infection conditions, we chose to focus on a subset of 650 genes consisting in transcripts showing a strain-specific regulation with an absolute value of the log_2_ ratio equal or more than 3.0, on which we performed a detailed functional annotation (Additional file 2). We performed a classification of all these differentially expressed *Bd* genes into 18 categories (Table 2).

Beside genes encoding miscellaneous and unknown proteins which were the most numerous, genes related to defense-associated proteins and hormonal synthesis and response, were well represented. Particularly, the defense response class contained a putative MLA6-like encoding gene solely induced during infection by *Fg*don^+^ strain (Additional file 2). The *Mla6* gene belongs to the barley *Mla* (mildew-resistance locus A) *R* locus [33]. Given, the specific induction pattern of the corresponding gene in our study, one could hypothesize that the MLA6-like protein might function as a DON receptor. JA biosynthesis and signalling were shown to be induced upon infection by both *Fg* strains. A putative *LOX* gene and a gene potentially encoding a 12-oxophytodienoate reductase, both belonging to the JA pathway [34], were specifically and strongly induced by the *Fg*don^+^ strain, indicating that oxylipins are important players in the interaction of toxicogenic strains of *Fg* with the susceptible host *Bd* ecotype Bd21. Moreover, four genes encoding putative ZIM domain containing (JAZ) proteins related to JAI3 [35] were also differentially induced, three of which (*Bradi3g23180*, *Bradi3g23190* and *Bradi1g72590*) being specifically up-regulated by the *Fg*don^+^ strain. In tomato, JAZ proteins regulate the progression of cell death in response to the phytotoxin coronatine produced by *Pseudomonas syringae* pv. *tomato* DC3000 [62]. Whether DON interferes directly with the JA pathway as coronatine by suppressing the plant defense responses would deserve attention.

A number of ET-responsive transcription factor-encoding genes appeared to be induced upon infection and particularly the AP2/EREB family of transcription factors, for which induction was mainly observed after *Fg*don^+^ infection (Additional file 2). Among these, the most induced was the *Bradi2g25050* gene. There are 132 genes encoding the AP2/EREB superfamily in the *Bd* genome ( http://bioinfo.bti.cornell.edu/cgi-bin/itak/index.cgi). *Bradi2g25050* is homologous to the Arabidopsis *AP2/EREB* gene *At1g22810* induced by 12-oxophytodienoic acid, a precursor of JA [63]. The induction of specific elements of the JA/ET pathways clearly appears to be a hallmark of DON production in *Bd* infected with *Fg*. JA and ET are generally accepted as molecules involved in the defense response towards necrotophic pathogens [64]. Our results suggest that at the time point considered for the transcriptome analysis (96 hai), *Fg* behaves as a necrotroph on *Bd* spikes. Such findings are in accordance with previous results obtained in wheat, indicating that the fungus most likely behaves as a hemibiotroph, growing intercellularly at the front of colonization without altering the viability of plant cells at early steps of the infection process then inducing cell death in more colonized tissues [65]. The involvement of DON in the switch to necrotrophy, although likely, remains unclear. Similarly, Ding et al. [66] found that both JA and ET biosynthesis and signalling were specifically induced in late time points of the infection of the resistant wheat cultivar Wangshuibai they used in their work. JA and ET signalling were also involved in the induction of several defense genes in the resistant wheat cultivar Dream [15].

Genes encoding proteins with antioxidant functions or involved in the redox balance including a putative ascorbate oxidase and glutaredoxins, were retrieved from our data (Table 2, Additional file 2). Most of these genes exhibit a higher induction following infection by the *Fg*don^+^ strain. In wheat, DON production during infection has been shown to induce ROS, which could be involved in promoting fungal necrotrophic growth [67]. Our results suggest that a similar process occurs in *Bd* following infection by a *Fg* toxicogenic strain.

Detoxification of xenobiotics in plants involves chemical modifications by enzymes such as UGTs, GSTs or CYPs and further steps of transport or compartmentation [10]. DON detoxification has frequently been proposed as one of the resistance mechanisms of small grain cereals to FHB [68]. Among the detoxification processes, glucosylation of DON into DON-3Glc is the most documented mechanisms [8, 69]. Glutathionylation has been proposed as an alternative detoxification process [13] and glutathione-conjugates of DON have been recently detected in DON-treated wheat spikes [70], indicating that plants may process the mycotoxin *via* various detoxification pathways. As expected in the design of our experiments, a number of genes potentially involved in detoxification processes were identified among the genes differentially regulated between the two infection treatments. These include genes encoding putative UGTs and GSTs suggesting that DON detoxification mechanisms in *Bd* are very similar to those observed in wheat and barley. Most interestingly, the most induced UGT-encoding gene in our data does correspond to the *Bradi5g03300* gene, recently shown to be involved in DON glucosylation [40], further reinforcing this mechanism as a major DON detoxification process in *Bd*. Analysis of the 8 GST-encoding genes exhibiting differential induction by the two *Fg* strains also pointed out exciting results. Among these, 5 belong to a cluster of 16 GST-encoding genes on chromosome 3. In this cluster, the *Bradi3g31860* gene is an interesting candidate for further studies on DON detoxification as it is specifically induced by the *Fg*don^+^ strain.

Metabolomic analyses of the interaction of barley with *Fg* have allowed the identification of constitutive or induced resistance-related metabolites belonging to different chemical groups, fatty acids, phenylpropanoids and flavonoids, and confirmed that DON-3Glc is a robust resistance indicator metabolite [16, 17, 71, 72]. Two metabolomic studies have been performed in *Bd* in interaction with the fungal pathogen *M. grisea* [73, 74]. In Parker et al. [74], in particular, targeted metabolite profiling using GC-MS showed that a metabolic re-programming occurred upon fungal infection in *Bd* and that it was similar to what observed in two other host plants, rice and barley. No secondary metabolite profiling was performed so far in *Bd* interacting with *Fg*. Our metabolite profiling analysis confirmed our transcriptome data, and especially the involvement of the Trp pathway in *Bd* response to *Fg* infection. Most interestingly, the most up-regulated metabolites were Trp and Trp-derived compounds as tryptamine, serotonin, Fer-serotonin and Cou-serotonin. Trp-derived secondary metabolites have been described as mediating the outcome of plant-pathogen interactions in cereals [75], and genes encoding enzymes involved in the biosynthesis of tryptophan and tryptamine have been shown to be up-regulated in the barley/*Fg* interaction [12]. The most widely distributed ones are benzoxazinoids which have been shown to be produced by maize, rye, wheat and wild barley species [76]. A recent phylogenetic study of the benzoxazinoid biosynthetic pathway of Poaceae suggested that, as for rice and *Setaria italica*, *Bd* would not produce benzoxazinoids [77]. Our results show that, similarly to rice, *Bd* produces other indole-derived compounds, such as serotonin and its hydroxycinnamic acid conjugates, Cou-serotonin and Fer-serotonin. In rice, serotonin has been shown to delay leaf senescence [78] and to be involved in defense responses against *Bipolaris oryzae*, the causal agent of rice brown spot [38]. More recently, this secondary metabolite was postulated to be a novel wheat phytoalexin and shown to strongly inhibit the sporulation of the necrotrophic wheat pathogen *S. nodorum* [79]. Here we show that serotonin has a slight but significant negative effect on *Fg-in vitro* radial growth and conidia germination. Our results need to be completed to determine the precise role of serotonin in the *Bd*/*Fg* interaction as our metabolite profiling was performed on soluble metabolites, but it may be involved in the restriction of fungal growth through incorporation into cell walls, as already shown in rice [38, 42].

## Conclusion

The objective of this work was to gain insight, using high throughput methods, into the possibility of using *Bd* as a good model plant species for studying the interaction with *Fg*, the causal agent of Fusarium head blight. We showed that *Fg* uses its main mycotoxin, DON, as a virulence factor. Our transcriptomic data are reminiscent of what was previously found in wheat and barley. Particularly, the JA- and ET-signalling pathways are important for the plant response to the pathogen. Moreover, the resistance mechanisms to *Fg* and particularly to DON, involve multiple mechanisms and especially those related to detoxification of xenobiotics similar to what has been described in other monocots, with the exception of benzoxazenoids which appear not to be produced by *Bd*. Altogether, our results show that *Bd* exhibits many defense hallmarks to *Fg* reminiscent of those already identified in cereal crop species. Together with its amenability in laboratory conditions, this makes this plant species a very good model to study cereal resistance mechanisms towards the major disease FHB.

## Electronic supplementary material

Additional file 1: **List and efficiency of primer pairs used in qPCR experiments.** (DOCX 21 KB)

Additional file 2: **Functional annotation of genes exhibiting an absolute value of log**_**2**_ **ratio ≥ 3.0 between** ***Fg*** **DON** ^**+**^ **(PHI_WT) and** ***Fg*** **DON** ^**-**^ **(PHI_dTri)-infected spikelets.** (XLSX 87 KB)

Additional file 3: **Fold change in accumulation of six** ***B. distachyon*** **defense-related genes following inoculation with** ***F. graminearum.*** A: Genes for which expression is more induced following infection by the *Fg* don ^+^ strain than by the *Fg* don ^-^ strain. B: Genes for which expression is more induced following infection by the *Fg* don ^-^ strain than by the *Fg* don ^+^ strain. qRT-PCR was performed on samples from the *B. distachyon* Bd21 ecotype at 0, 24, 48, 72, 96 and 168 hai with either the *Fg* don ^+^ (black bars) or the *Fg* don ^-^ (white bars) strain. The relative quantity of target gene transcripts was calculated using the comparative cycle threshold method (2^-ΔΔCt^). The infected samples were quantified relatively to the Tween-treated controls at the same time points. The *B. distachyon UBC18* gene (*Bradi4g00660*) was used as an endogenous control to normalize the data for differences in input RNA between the different samples. Data represent mean values of three independent biological experiments, error bars are representing the standard deviation. (TIFF 79 KB)

Additional file 4: **Functional annotation and transcriptional regulation of genes encoding enzymes potentially involved in** ***B. distachyon*** **tryptophan pathway following mock- (Tween),** ***Fg*** **DON** ^**+**^ **- (PHI_WT) or** ***Fg*** **DON** ^-^ **- (PHI_dTri) inoculation.** (DOCX 17 KB)

Additional file 5: **Description of the different peaks identified in HPLC-MS chromatogram.** (DOCX 17 KB)

Additional file 6: **Absolute quantification of serotonin, tryptophan and tryptamine in spikelets 96 h after infection by** ***F. graminearum Fg*** **DON** ^**+**^ **and** ***Fg*** **DON** ^**-**^ **strains.** Data represent mean values of three independent biological experiments, error bars are representing the standard deviation. Letters indicate the significance of the difference between conditions (*t* test p-value ≤ 0.02). (TIFF 54 KB)
