# Supplementary material for: Differential gene expression and metabolomic analyses of Brachypodium distachyon infected by deoxynivalenol producing and non-producing strains of Fusarium graminearum
Source: BMC Genomics. 2014 Jul 25;15(1):629. doi: 10.1186/1471-2164-15-629 (PMC4124148; doi:10.1186/1471-2164-15-629)
Supplement: Supplementary file 1 — Additional file 1: List and efficiency of primer pairs used in qPCR experiments. (DOCX 21 KB) [file 12864_2014_6327_MOESM1_ESM.docx]

**Additional file 1. List of primers used in qPCR experiments**

| **Gene** | **Nucleotide sequence** | **Polarity** | **Putative fonction** | **Amplicon length (bp)** |
| --- | --- | --- | --- | --- |
|  |  |  |  |  |
| *Bd4g00660* | 5'-ACCCTCTACGCTGGTGAGAC-3' | Forward | UBC18 (plant reference gene) | 118 |
|  | 5'-TTGCTGTAAATGTGCGGATG-3' | Reverse |  |  |
| *Bd1g75310* | 5'-GCTACGTCTTCAGGCACTCC-3' | Forward | Cytochrome monoxygenase (CYP711A29) | 103 |
|  | 5'-CGATCGATGACTTGGAGCTT-3' | Reverse |  |  |
| *Bd3g22880* | 5'-AGCAATGAGGATGAAGTGG-3' | Forward | Transpoter MATE efflux | 100 |
|  | 5'-GGACACCTGAAAGAGAAGTATG-3' | Reverse |  |  |
| *Bd2g41010* | 5'-GGTGGGTGCGAGTCTACAAT-3' | Forward | Unkown | 169 |
|  | 5'-CAGGAAGGCTTGGAGTGGT-3' | Reverse |  |  |
| *Bd2g35930* | 5'-CCCACTTCGGGTTCACATAC-3' | Forward | Gluthatione S-transferase GSTF1-like | 147 |
|  | 5'-TCAGCACGAGGTTCATCATC-3' | Reverse |  |  |
| *Bd4g39950* | 5'-CGCACTAAAGAGGTGCTTCC-3' | Forward | NADP-dependent alkenal double bond reductase P1-like | 179 |
|  | 5'-CGACGAGGCAGAATAGGTTC-3' | Reverse |  |  |
| *Bd4g05040* | 5'-CCAAGCTCACGGTGGAGTAT-3' | Forward | PR10 | 108 |
|  | 5'-CCTCGACCTTCTTGAGCAAC-3' | Reverse |  |  |
| *Bd4g36870* | 5'-GATCGACGACGGGCTCTAC-3' | Forward | Ornithine decarboxylase | 116 |
|  | 5'-TCGAGGCGTACGTCTTCTCT-3' | Reverse |  |  |
| *Bd4g36810* | 5'-GAACCACCCACATCCAAG-3' | Forward | Ornithine decarboxylase | 108 |
|  | 5'-GCAACAGAGCACCTACATTT-3' | Reverse |  |  |
| *Bd1g50067* | 5'-AACGACTACCGCAACCTCAT-3' | Forward | Arginine decarboxylase | 114 |
|  | 5'-GAGGACCCCTTCCTTGAACT-3' | Reverse |  |  |
| *Bd3g47110* | 5'-CCAAACAATTAAGGAGATCAATTAGAA-3' | Forward | Phenylalanine ammonia-lyase | 167 |
|  | 5'-CCCGAATACTGGAAAGTAAGATACA-3' | Reverse |  |  |
| *Bd3g53227* | 5'-AGGGTGCACTAGCTGGAAGA-3' | Forward | 3-hydroxy-3-methyl-glutaryl-CoA reductase (HMGR) | 129 |
|  | 5'-TGTCGGGGAAGTTATTCTGC-3' | Reverse |  |  |
| *Bd2g13360* | 5'-GAGTGGGAGTCATTCGGAAA-3' | Forward | Mevalonate kinase | 123 |
|  | 5'-CATGCTTCCGTAAGTGCTGA-3' | Reverse |  |  |
| *Bd1g12360* | 5'-AAGGGGCGGATGTATGTA-3' | Forward | PR1-like | 64 |
|  | 5'-CACACGAAAGGAACAGCA-3' | Reverse |  |  |
| *Bd5g14430* | 5'-CGGGCTACTACAGGGACT-3' | Forward | Chitinase | 109 |
|  | 5'-TAAGACACGCAGGCATCT-3' | Reverse |  |  |
| *Bd2g26810* | 5'-GCAAGGGTTCAAGTATGAGA-3' | Forward | Chitinase | 102 |
|  | 5'-ACACCATCACCACCAACTC-3' | Reverse |  |  |
| *Bd2g02150* | 5'-TACCCTCTCAAGCAGTTCA-3' | Forward | PR6 | 100 |
|  | 5'-TAGCCAGTAAACCCCAAT-3' | Reverse |  |  |
| *Bd1g39190* | 5'-TCCGACCAGGCTCTCTAC-3' | Forward | PR9 | 125 |
|  | 5'-GGTATGTTCCCCATCTTGAC-3' | Reverse |  |  |
| *Bd3g44910* | 5'-ACATCATGGGCGTCTCCTAC-3' | Forward | PR2 | 126 |
|  | 5'-ACGGGTAGCAGTTGATGAGG-3' | Reverse |  |  |

**Additional file 1 (continued)**

| **Gene** | **Nucleotide sequence** | **Polarity** | **Putative fonction and use** | **Amplicon length (bp)** |
| --- | --- | --- | --- | --- |
|  |  |  |  |  |
| *Bd2g04720* | 5'-CAAGCATTTGTCCTGTTTCCC-3' | Forward | UDP-glycosyltransferase | 170 |
|  | 5'-GTGGAAAAAGCAGTATGCACC-3' | Reverse |  |  |
| *Bd2g04760* | 5'-TGCTTGATACAGATGGTAGTAGGC-3' | Forward | UDP-glycosyltransferase | 246 |
|  | 5'-CAATGCAAAGGAGAGCGTCTA-3' | Reverse |  |  |
| *Bd5g03390* | 5'-GGAGAAGCAGACCATTTCATCTAT-3' | Forward | UDP-glycosyltransferase | 177 |
|  | 5'-TTGCATTTCGTTTTGAGTTCTT-3' | Reverse |  |  |
| *Bd5g03300* | 5'-AAGAAAAGCTTGAATGGACAACTAA-3' | Forward | UDP-glycosyltransferase | 138 |
|  | 5'-CGCGTGCATTGTCTCCTT-3' | Reverse |  |  |
| *Bd5g02780* | 5'-TCAACCAACTACAGCAAAA-3' | Forward | UDP-glycosyltransferase | 81 |
|  | 5'-CCTCTCTACCTCTTCCTTTC-3' | Reverse |  |  |
| *Bd5g03370* | 5'-ACCAACCAACTACTGCGA-3' | Forward | UDP-glycosyltransferase | 81 |
|  | 5'-CTCTCAATCTTTTCCCTTGT-3' | Reverse |  |  |
| *Bd5g03400* | 5'-TACAAAAGGAATGCTATGAA-3' | Forward | UDP-glycosyltransferase | 73 |
|  | 5'-AATACTTGGTGGCGAAAT-3' | Reverse |  |  |
| *Bd5g03380* | 5'-GGCGTCAACTTCTTCAGC-3' | Forward | UDP-glycosyltransferase | 128 |
|  | 5'-GAGTCGCACAGTCCATTTC-3' | Reverse |  |  |
| 18S *F. graminearum* | 5'-GTCCGGCCGGGCCTTTCC-3' | Forward | Ribosomal region 18S (fungal reference gene) | 68 |
|  | 5'-AAGTCCTGTTTCCCCGCCACGC-3' | Reverse |  |  |
