# Supplementary figures and images for: Differential gene expression and metabolomic analyses of Brachypodium distachyon infected by deoxynivalenol producing and non-producing strains of Fusarium graminearum
Source: BMC Genomics. 2014 Jul 25;15(1):629. doi: 10.1186/1471-2164-15-629 (PMC4124148; doi:10.1186/1471-2164-15-629)

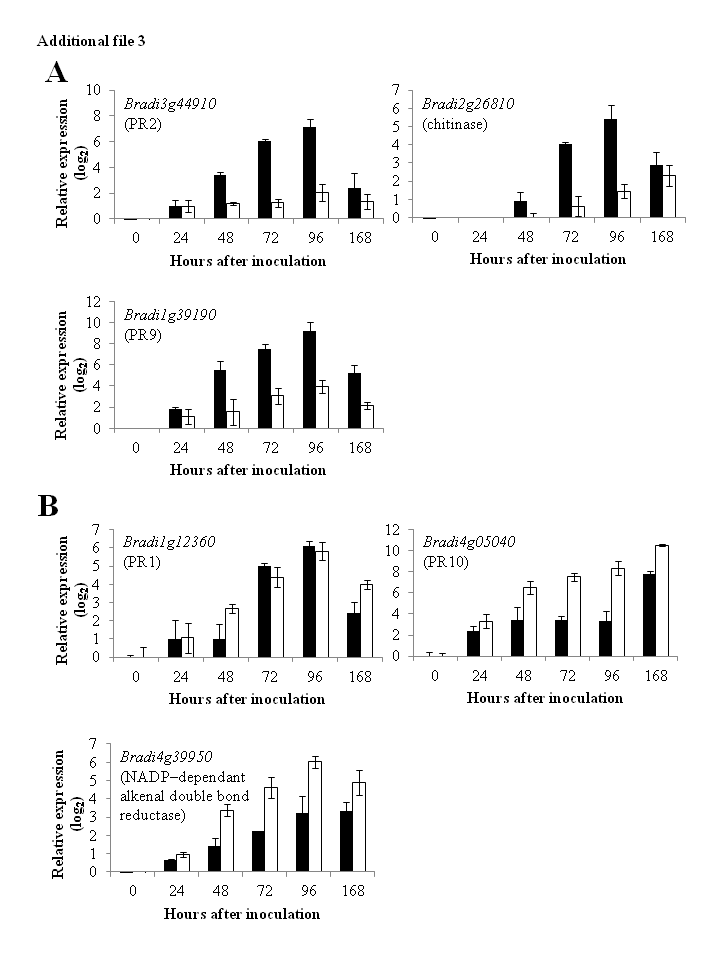

Supplement: Supplementary file 3 — Additional file 3: Fold change in accumulation of six B. distachyon defense-related genes following inoculation with F. graminearum. A: Genes for which expression is more induced following infection by the Fg don + strain than by the Fg don - strain. B: Genes for which expression is more induced following infection by the Fg don - strain than by the Fg don + strain. qRT-PCR was performed on samples from the B. distachyon Bd21 ecotype at 0, 24, 48, 72, 96 and 168 hai with either the Fg don + (black bars) or the Fg don - (white bars) strain. The relative quantity of target gene transcripts was calculated using the comparative cycle threshold method (2-ΔΔCt). The infected samples were quantified relatively to the Tween-treated controls at the same time points. The B. distachyon UBC18 gene (Bradi4g00660) was used as an endogenous control to normalize the data for differences in input RNA between the different samples. Data represent mean values of three independent biological experiments, error bars are representing the standard deviation. (TIFF 79 KB) [file 12864_2014_6327_MOESM3_ESM.tiff]

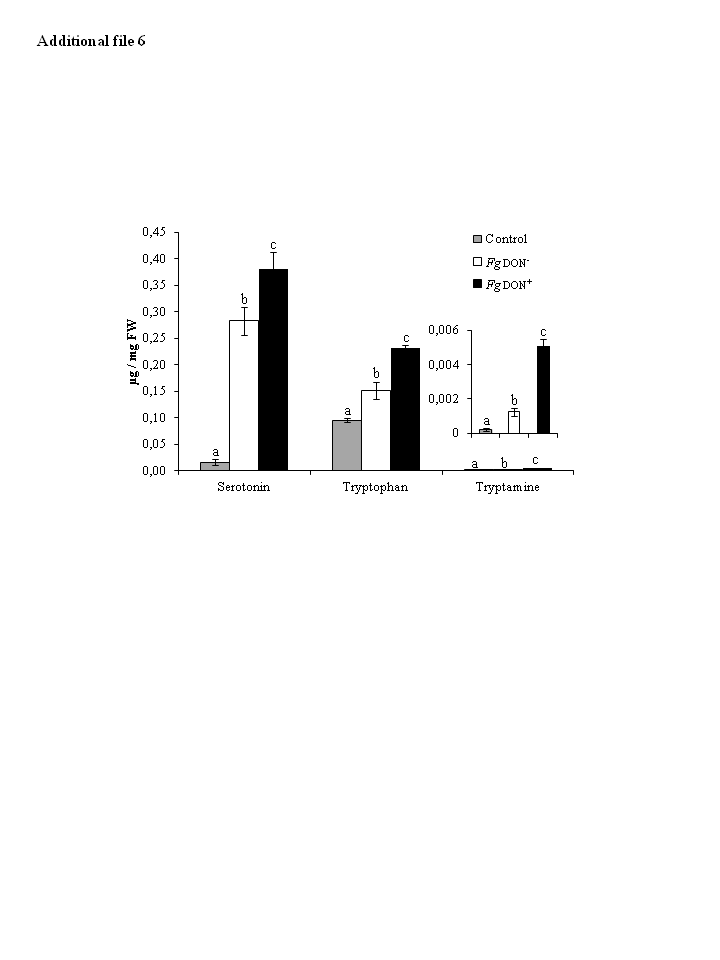

Supplement: Supplementary file 6 — Additional file 6: Absolute quantification of serotonin, tryptophan and tryptamine in spikelets 96 h after infection by F. graminearum Fg DON + and Fg DON - strains. Data represent mean values of three independent biological experiments, error bars are representing the standard deviation. Letters indicate the significance of the difference between conditions (t test p-value ≤ 0.02). (TIFF 54 KB) [file 12864_2014_6327_MOESM6_ESM.tiff]
