# Supplementary material for: Differential gene expression and metabolomic analyses of Brachypodium distachyon infected by deoxynivalenol producing and non-producing strains of Fusarium graminearum
Source: BMC Genomics. 2014 Jul 25;15(1):629. doi: 10.1186/1471-2164-15-629 (PMC4124148; doi:10.1186/1471-2164-15-629)
Supplement: Supplementary file 4 — Additional file 4: Functional annotation and transcriptional regulation of genes encoding enzymes potentially involved in B. distachyon tryptophan pathway following mock- (Tween), Fg DON + - (PHI_WT) or Fg DON - - (PHI_dTri) inoculation. (DOCX 17 KB) [file 12864_2014_6327_MOESM4_ESM.docx]

**Additional file 4:** Functional annotation and transcriptional regulation of genes potentially involved in the *B. distachyon* tryptophan pathway from KEGG information.

^a^Comparison between *Fg* don^+^ (PHI_WT) and *Fg* don^-^ (PHI_dTri)-infected spikelets.

^b^Comparison between *Fg* don^+^ (PHI_WT) strain- and mock (TWEEN)-inoculated spikelets.

^c^Comparison between *Fg* don^-^ (PHI_dTri) strain- and mock (TWEEN)-inoculated spikelets.

Induction is indicated by red backgrounds, repression by green backgrounds.

*Bonferroni p-value: a gene is declared differentially expressed if the Bonferroni p-value is less than 0.05.

| **Gene** | **PHIdTrivsPHI_WT^a^** | **Bonferroni*** | **TWEENvsPHI_WT^b^** | **Bonferroni*** | **TWEENvsPHI_dTri^c^** | **Bonferroni*** |  | **Functional annotation** |
| --- | --- | --- | --- | --- | --- | --- | --- | --- |
| Bradi1g35960 | -1.24 | 1.00E+0 | -2.26 | 3.11E-6 | -1.02 | 1.00E+0 |  | anthranilate phosphoribosyltransferase |
| Bradi1g76800 | 0.65 | 1.00E+0 | 3.88 | 0.00E+0 | 3.23 | 0.00E+0 |  | anthranilate phosphoribosyltransferase |
|  |  |  |  |  |  |  |  |  |
| Bradi4g08830 | 1.11 | 1.00E+0 | 7.22 | 0.00E+0 | 6.11 | 0.00E+0 |  | indole-3-glycerol phosphate synthase |
| Bradi5g05430 | -0.01 | 1.00E+0 | 4.26 | 0.00E+0 | 4.26 | 0.00E+0 |  | indole-3-glycerol phosphate synthase |
| Bradi5g12870 | -1.78 | 3.31E-3 | -2.12 | 4.29E-5 | -0.33 | 1.00E+0 |  | indole-3-glycerol phosphate synthase |
|  |  |  |  |  |  |  |  |  |
| Bradi1g05450 | 5.07 | 0.00E+0 | 8.49 | 0.00E+0 | 3.42 | 0.00E+0 |  | indole-3-glycerol phosphate lyase |
| Bradi1g35600 | -0.90 | 1.00E+0 | 4.91 | 0.00E+0 | 5.81 | 0.00E+0 |  | tryptophan synthase beta chain 2 |
| Bradi1g55440 | 0.46 | 1.00E+0 | 5.08 | 0.00E+0 | 4.63 | 0.00E+0 |  | indole-3-glycerol phosphate lyase |
| Bradi3g14490 | 1.03 | 1.00E+0 | 3.48 | 0.00E+0 | 2.45 | 0.00E+0 |  | tryptophan synthase beta chain 2 |
|  |  |  |  |  |  |  |  |  |
| Bradi3g14730 | 2.60 | 1.58E-10 | 8.37 | 0.00E+0 | 5.77 | 0.00E+0 |  | aromatic-L-amino-acid decarboxylase-like |
| Bradi3g14740 | 3.15 | 0.00E+0 | 9.59 | 0.00E+0 | 6.44 | 0.00E+0 |  | aromatic-L-amino-acid decarboxylase-like |
| Bradi3g14750 | 3.32 | 0.00E+0 | 7.72 | 0.00E+0 | 4.40 | 0.00E+0 |  | aromatic-L-amino-acid decarboxylase-like |
| Bradi3g14760 | 4.11 | 0.00E+0 | 7.34 | 0.00E+0 | 3.23 | 0.00E+0 |  | aromatic-L-amino-acid decarboxylase-like |
| Bradi3g14780 | 1.40 | 1.00E+0 | 6.42 | 0.00E+0 | 5.02 | 0.00E+0 |  | aromatic-L-amino-acid decarboxylase-like |
|  |  |  |  |  |  |  |  |  |
| Bradi4g39240 | 1.13 | 1.00E+0 | 4.98 | 0.00E+0 | 3.85 | 0.00E+0 |  | Cytochrome P450 (CYP71P1) |
