# Supplementary material for: Differential gene expression and metabolomic analyses of Brachypodium distachyon infected by deoxynivalenol producing and non-producing strains of Fusarium graminearum
Source: BMC Genomics. 2014 Jul 25;15(1):629. doi: 10.1186/1471-2164-15-629 (PMC4124148; doi:10.1186/1471-2164-15-629)
Supplement: Supplementary file 5 — Additional file 5: Description of the different peaks identified in HPLC-MS chromatogram. (DOCX 17 KB) [file 12864_2014_6327_MOESM5_ESM.docx]

**Additional file 5**: Description of the different peaks identified in HPLC-MS chromatogram

| Peak n° | Retention time (min) | max UV (nm) | Observed Mass (Da) | Compounds | Arbitral units | | |
| --- | --- | --- | --- | --- | --- | --- | --- |
|  |  |  |  |  | Control | *Fg* don^-^ | *Fg* don^+^ |
| 1 | 1.85 | 304 | 351.1871 | Unknown | 0^a^ | 58.22 ± 14.6^b^ | 108.4 ± 30.9^b^ |
| 2 | 3.2 | 275, sh 298 | 177.1028 | Serotonin* | 0^a^ | 135.7 ± 28.2^b^ | 213.6 ± 18.7^c^ |
| 3 | 3.95 | 248, 295 | 439.0986 | Unknown | n. q. | n. q. | n. q. |
| 4 | 5.65 | 280 | 205.0995 | Tryptophan* | 44.4 ± 6.1^a^ | 73.6 ± 7.8^b^ | 105.0 ± 4.7^c^ |
| 5 | 6.64 | 280 | 161.1073 | Tryptamin* | n. d | n. d | n. d |
| 6 | 7.5 | sh 295, 324 | 369.1197 | Feruloyl quinic acid | 4.4 ± 1.6^a^ | 6.4 ± 2.9^a^ | 10.3 ± 1.9^b^ |
| 7 | 8.63 | sh 296, 312 | 339.1083 | Coumaroyl quinic acid | n. q. | n. q. | n. q. |
| 8 | 8.9 | sh 295, 310 | 437.1068 | Cinnamic acid derivative | 11.4 ± 1.3^a^ | 10.8 ± 0.9a | 3.8 ± 1.4^b^ |
| 9 | 9.15 | sh 296, 325 | 369.1197 | Cinnamic acid derivative | 13.1 ± 3.7 | 14.3 ± 2.3 | 20.1 ± 3.9 |
| 10 | 9.4 | 270, 348 | n. d | Flavonoid glycoside | n. q. | n. q. | n. q. |
| 11 | 9.5 | 270, 348 | 581.1608 | Flavonoid glycoside | n. q. | n. q. | n. q. |
| 12 | 9.8 | 270, 350 | 611.1719 | Flavonoid glycoside | n. q. | n. q. | n. q. |
| 13 | 10.3 | 270, 335 | 565.1651 | Flavonoid glycoside | n. q. | n. q. | n. q. |
| 14 | 10.6 | 271, 350 | 595.1771 | Flavonoid glycoside | n. q. | n. q. | n. q. |
| 15 | 10.8 | 270, 346 | 595.1773 | Flavonoid glycoside | n. q. | n. q. | n. q. |
| 16 | 11.3 | 270, 345 | 621.2271 | Flavonoid glycoside | n. q. | n. q. | n. q. |
| 17 | 13.85 | 292, 306 | 323.1401 | *p-*coumaroyl serotonin | 0^a^ | 3.8 ± 2.2^b^ | 28.0 ± 7.8^c^ |
| 18 | 14.4 | 294, 314 | 353.1508 | Feruloyl serotonin | 0^a^ | 0^a^ | 9.2 ± 3.4^b^ |

Different letters indicate significant differences between conditions; t test, p-value ≤ 0.02.

Asterisks indicate compounds identified using a standard; n.q. = not quantified
